# Letter to Editor: “Discrepant Results on Two Different Single Gene Non-invasive Prenatal Tests for Cystic Fibrosis: A Case Report”

**DOI:** 10.1055/a-2946-0807

**Published:** 2026-09-18

**Authors:** Shahram S. Daneshmand

**Affiliations:** 1 Department of Maternal-Fetal MedicineScripps Clinic Medical GroupSan DiegoCaliforniaUnited States

## 



Dr. Hogg and colleagues present a clinically important case highlighting the complexities of single-gene noninvasive prenatal testing (sgNIPT) for cystic fibrosis. A known carrier couple received a “Decreased Risk” result (1/51) on the Unity platform (BillionToOne) at 11 weeks, followed by a high-risk result on another sgNIPT platform that was confirmed by amniocentesis as homozygous F508del. This case usefully illustrates both the promise and limitations of these emerging tests.

We wish to highlight a key disconnect. In clinical practice, Unity uses a four-tier reporting system (High Risk, Increased Risk, Decreased Risk, Low Risk). However, the primary validation studies collapsed into binary categories using varying thresholds (≥1/4 or >1/100 as screen-positive).

Consequently, the “Decreased Risk” result of 1/51 in this case would have been classified differently under those study definitions. Published performance metrics may therefore not map directly onto the commercial reports clinicians actually receive.

This raises an important question about clinical interpretation: how reliably can published validation metrics inform practice when the analytic framework differs from real-world reporting? Clinicians may reasonably assume published sensitivity and specificity apply to the exact categories they discuss with patients, which is not the case.

We commend Dr. Hogg for underscoring sgNIPT limitations and reinforcing diagnostic testing as the gold standard. Better alignment between study methodologies and commercial reporting would greatly improve transparency and clinical confidence.

